# Marine Bacterial Aromatic Polyketides From Host-Dependent Heterologous Expression and Fungal Mode of Cyclization

**DOI:** 10.3389/fchem.2018.00528

**Published:** 2018-10-30

**Authors:** Chunshuai Huang, Chunfang Yang, Yiguang Zhu, Wenjun Zhang, Chengshan Yuan, Changsheng Zhang

**Affiliations:** ^1^CAS Key Laboratory of Tropical Marine Bio-resources and Ecology, Guangdong Key Laboratory of Marine Materia Medica, South China Sea Institute of Oceanology, RNAM Center for Marine Microbiology, Chinese Academy of Sciences, Guangzhou, China; ^2^University of Chinese Academy of Sciences, Beijing, China

**Keywords:** heterologous expression, aromatic polyketides, type II polyketide synthase, cyclization modes, pathway crosstalk

## Abstract

The structure diversity of type II polyketide synthases-derived bacterial aromatic polyketides is often enhanced by enzyme controlled or spontaneous cyclizations. Here we report the discovery of bacterial aromatic polyketides generated from 5 different cyclization modes and pathway crosstalk between the host and the heterologous fluostatin biosynthetic gene cluster derived from a marine bacterium. The discovery of new compound SEK43F (**2**) represents an unusual carbon skeleton resulting from a pathway crosstalk, in which a pyrrole-like moiety derived from the host *Streptomyces albus* J1074 is fused to an aromatic polyketide SEK43 generated from the heterologous fluostatin type II PKSs. The occurrence of a new congener, fluoquinone (**3**), highlights a bacterial aromatic polyketide that is exceptionally derived from a characteristic fungal F-mode first-ring cyclization. This study expands our knowledge on the power of bacterial type II PKSs in diversifying aromatic polyketides.

## Introduction

Aromatic polyketides (APKs) comprise a rich class of natural products with diverse structures and exhibit antimicrobial, antitumor, antiparasitic, antiviral, and other activities (Shen, 2000; Hertweck et al., 2007; Das and Khosla, 2009; Zhou et al., 2010; Zhang Z. et al., 2017). Most bacterial APKs are synthesized by type II polyketide synthases (PKSs). The “minimal” type II PKSs consist of a set of iteratively used enzymes including two ketosynthase units (KS_α_ and KS_β_) and an acyl-carrier protein (ACP) (Shen, 2000; Hertweck et al., 2007; Das and Khosla, 2009; Zhou et al., 2010; Zhang Z. et al., 2017). The KS_α_ unit catalyzes iterative decarboxylative condensations of ACP-tethered malonyl to generate a linear poly-β-ketone chain, the length of which is controlled by the KS_β_ unit (also known as chain-length factor, CLF). The poly-β-ketone chains are converted into diverse APKs by enzymatic tailoring modifications such as cyclization, oxidation, glycosylation, and methylation. In many cases, shunt products are also produced from the reactive poly-β-ketone chains by spontaneous and aberrant cyclization to further enhance the structural diversity of APKs (Shen, 2000; Hertweck et al., 2007; Das and Khosla, 2009; Zhou et al., 2010; Zhang Z. et al., 2017).

Fluostatins (FSTs) are a growing family of atypical angucycline-type APKs and display antibacterial, antitumor and peptidase inhibition activities (Akiyama et al., 1998a,b; Baur et al., 2006; Feng et al., 2010; Zhang et al., 2012; Yang et al., 2015; Jiang et al., 2017; Zhang W. et al., 2017; Huang et al., 2018; Jin et al., 2018). Recently, heterologous expression of the type II PKS gene cluster (*fls*) of FSTs from South China Sea-derived *Micromonospora rosaria* SCSIO N160 in *Streptomyces coelicolor* YF11 (Zhou et al., 2012) enabled the production of new FST derivatives (Yang et al., 2015). Intriguingly, introduction of the *fls*-gene cluster in *Streptomyces albus* J1074 led to production of diverse C–C and C–N coupled dimeric FSTs (Huang et al., 2018). Herein we report the discovery of APKs derived from different chain lengths and cyclization patterns in the heterologous host *S. albus* J1074.

## Materials and methods

### General experimental procedures

Optical rotation was determined on a 341 polarimeter (Perkin Elmer, Inc.). UV spectrum was recorded with a U-2900 spectrophotometer (Hitachi). IR spectrum was obtained using a Nicolet^*^6700 FT-IR spectrometer (Thermo Scientific). ^1^H, ^13^C, and 2D NMR spectra were recorded on Bruker 700 spectrometer with tetramethylsilane (TMS) as the internal standard. Low-resolution mass spectrometric data were determined using an amaZon SL ion trap mass spectrometer. High-resolution electrospray ionization mass spectrometric (HRESIMS) data were measured on a MaXis 4G UHR-TOFMS spectrometer (Bruker Daltonics Inc.). Column chromatography (CC) was performed with silica gel (100–200 mesh, Jiangyou Silica Gel Development, Inc., Yantai, P. R. China). Thin layer chromatography (TLC, 0.1–0.2 or 0.3–0.4 mm) was conducted with precoated silica gel GF254 (10–40 nm, Yantai) glass plates. Preparative TLC (pTLC) was conducted with precoated glass plates (silica gel GF254, 10–40 nm). Sephadex LH-20 (40–70 μm; Amersham Pharmacia Biotech AB, Uppsala, Sweden), and YMC^*^gel ODS-A (12 nm S-50 μm; Japan). MCI gel CHP-20P (75–150 μm, Mitsubishi Chemical Corp., Tokyo, Japan). Medium pressure liquid chromatography (MPLC) was performed on automatic flash chromatography (Cheetahtmmp 200, Bonna-Agela Technologies Co., Ltd.) with the monitoring wavelength at 220 nm and the collecting wavelength at 254 nm. Semi-preparative HPLC was carried out on a Hitachi-L2130 HPLC (equipped with a Hitachi L-2455 diode array detector) using a Phenomenex Luna C18 column (250 × 10 mm, 5 μm). Analytical HPLC was performed on an Agilent 1260 Infinity series instrument (equipped with a quaternary pump, a vacuum degasser, an autosampler, a thermostatic column compartment, and a DAD detector) using an Agilent ZORBAX SB-C18 column (150 × 4.6 mm, 5 μm) under the following program: solvent system (solvent A, 10% acetonitrile in water supplementing with 0.08% formic acid; solvent B, 90% acetonitrile in water); 5% B to 80% B (linear gradient, 0–20 min), 80% B to 100% B (20–21 min),100% B (isocratic elution, 21–24 min), 100% B to 5% B (24–25 min), 5% B (isocratic elution, 25–30 min) with the monitoring wavelength at 400 nm. Small scale production process was performed in a compact sterilizable-in-place fermentation system BioFlo 510 fermentator (Eppendorf AG, German).

### Bacterial material

The recombinant strain *S. albus* J1074/pCSG5033 has been previously described (Huang et al., 2018).

### Fermentation, extraction, and isolation

The recombinant strain *S. albus* J1074/pCSG5033 was cultured in the seed medium (3% tryptic soya broth, pH 7.0) and carried on a rotary shaker (200 rpm) at 28°C for 1–3 days. A 20 L scale fermentation was performed by inoculating 1.5 L of the seed culture into a 40 L fermentator (stirring rate:120–230 rpm; dissolved oxygen: 30%; ventilation volume: 15–20 L min^−1^; pH: 7.2–7.4; pressure: 3 psi; fermentation temperature: 28°C) containing 20 L of the production medium (0.1% peptone fish, 1% starch soluble, 0.6% corn powder, 0.2% bacterial peptone, 0.5% glycerol, 0.2% CaCO_3_, 3% sea salt), and cultured at 28°C for 3–5 days. A total of 20 L fermentation cultures were harvested and centrifuged to supernatants and mycelium. The supernatants and mycelium were extracted 3 times with equal volume of butanone and acetone, respectively. And then both were evaporated to dryness under reduced pressure and combined to obtain the crude extract (10.0 g).

The crude extract was subjected to normal phase silica gel (100–200 mesh) column chromatography eluted with a gradient solvent system of chloroform/methanol (from 100:0 to 0:100, v/v) to give four fractions (Fr.1–Fr.4) on the basis of initial assessment by thin-layer chromatography (TLC). Fr.1 was subjected to Sephadex LH-20 column chromatography, eluting with CHCl_3_/MeOH (1:1) to afford four subfractions (Fr.1.L1–Fr.1.L4). Subfraction Fr.1.L3 was further purified using C18 reversed phase MPLC [40 × 2.5 cm ID, eluting with a linear gradient of H_2_O/CH_3_CN (100:0 → 0:100, 15 mL min^−1^, 200 min)], Sephadex LH-20 column chromatography, and reversed-phase semi-preparative HPLC (H_2_O/CH_3_CN) successively, to yield **3** (7.0 mg), **4** (1.4 mg), **5** (9.6 mg), and **6** (7.0 mg). Subfraction Fr.2 was further separated with MCI gel column (CH_3_CN/H_2_O, from 0:100 to 100:0) to give five fractions Fr.2.M1-Fr.2.M5. SEK43F (**2**, 4.0 mg) was obtained from Fr.2.M3 by Sephadex LH-20 column chromatography and pTLC (petroleum ether/acetone 50:50), successively. FST C (**1**, 56.0 mg) was obtained from subfraction Fr.4.

#### SEK43F (2)

Yellow-green powder; UV (MeOH) λ_max_ (log ε) 452 (4.21), 296 (3.65), 257 (3.37), 205 (4.07) nm; IR ν_max_ 3,273, 1,593, 1,435, 1,287 cm^−1^; ^1^H and ^13^C NMR spectroscopic data, Table 1; HRESIMS *m/z* 486.1557 [M - H]^−^ (calcd for C_28_H_24_NO_7_, 486.1558).

**Table 1:** ^1^H (700 MHz) and ^13^C NMR (176 MHz) data for **2** and **3** in DMSO-*d*_6_ (δ in ppm).

**2**			**3**		

**Position**	δ_C_**, type**	δ_H_**, mult. (*****J*** **in Hz)**	**Position**	δ_C_**, type**	δ_H_**, mult. (*****J*** **in Hz)**
1	165.5, C		1	166.8, C
2	104.8, C		3	74.0, CH	5.02, m
3	180.8, C		4	31.8, CH_2_	4.12, dd (2.9, 18.1)
4	109.0, CH	5.59, s			3.31, dd (11.8, 18.2)
5	164.5, C			
6	36.9, CH_2_	3.58, s		
7	132.7, C		4a	146.8, C
8	121.6, CH	6.83, d (8.2)	5	114.6^†^, CH	7.61, s
9	130.6, CH	7.24, dd (8.2, 8.8)	5a	139.3, C
10	115.2, CH	6.82, d (8.8)	6	181.9, C
11	154.3, C		6a	132.8, C
12	131.3, C		7	118.9, CH	7.70, d (7.3)
13	200.3, C		8	136.6, CH	7.79, dd (7.3, 8.3)
14	116.1, C		9	124.9, CH	7.40, d (8.3)
15	165.6, C		10	161.7, C
16	101.2, CH	6.11, d (2.3)	10a	117.2, C
17	163.7, C		11	188.6, C
18	112.1, CH	6.07, d (2.3)	11a	114.9, C
19	143.5, C		12	165.0^†^, C
20	22.0, CH_3_	1.86, s	12a	121.0^†^, C
1'	134.9, CH	7.84, s	13	47.4, CH_2_	3.09, dd (5.0, 17.4)
2'	130.9, C				3.13, dd (7.4, 17.4)
3'	140.6, C			
4'	126.3, C			
5'	149.6, C		14	205.4, C
7'	10.3, CH_3_	2.23, s	15	30.4, CH_3_	2.20, s
8'	9.4, CH_3_	2.00, s	10-OH		12.60, s
9'	13.5, CH_3_	2.42, s		
11-OH		9.84, s		
15-OH		12.69, s		
17-OH		10.40, brs		
NH-6'		14.65, s		

† *Chemical shift observed in HSQC or HMBC spectra*.

#### Fluoquinone (3)

Yellow powder; [α] + 1.6 (*c* 0.22, CHCl_3_); UV (MeOH) λ_max_ (log ε) 414 (2.49), 275 (3.02), 253 (3.00), 227 (3.19), 203 (3.30) nm; IR ν_max_ 2,922, 1,686, 802 cm^−1^; ^1^H and ^13^C NMR spectroscopic data, Table 1; HRESIMS *m/z* 365.0672 [M - H]^−^ (calcd for C_20_H_13_O_7_, 365.0667).

### Biological assays

Antimicrobial activities were measured against seven indicator strains, *Staphylococcus aureus* ATCC 29213, *Enterococcus faecalis* ATCC 29212, *Escherichia coli* ATCC 25922, *Acinetobacter baumannii* ATCC 19606, *Bacillus subtilis* SCSIO BS01, *Micrococcus luteus* SCSIO ML01, and methicillin resistant *S. aureus* ATCC 43300, by previously described broth microdilution method (Yang et al., 2015). MCF7 (human breast adenocarcinoma cell line), NCI-H460 (human non-small cell lung cancer cell line), HepG2 (Human hepatocellular liver carcinoma cell line), and SF268 (human glioma cell line) were kindly used for cytotoxic activities assay *in vitro* according to the previously established protocols (Mosmann, 1983).

## Results and discussion

### Isolation and structure elucidation

**Figure 1:**
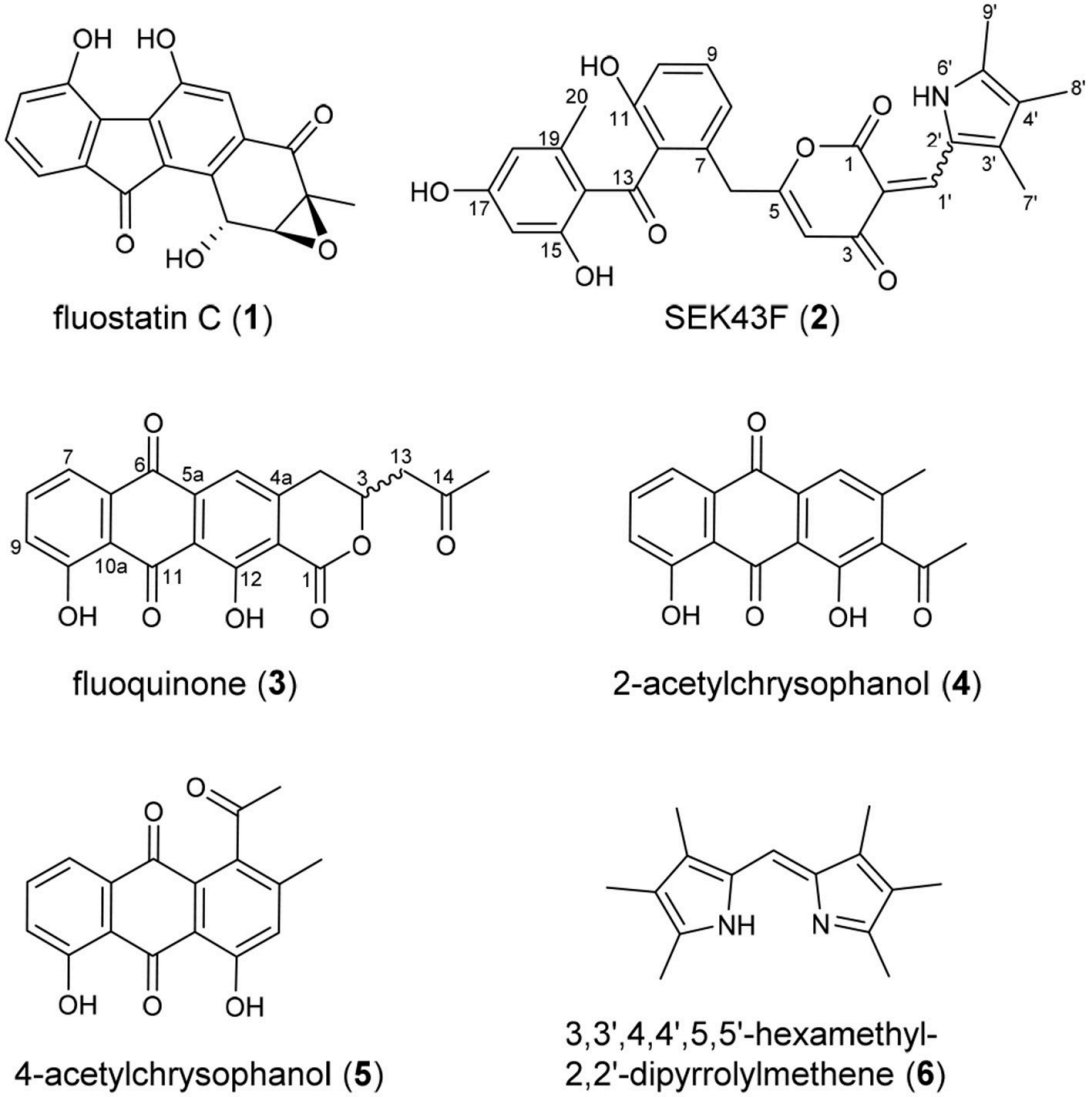
Chemical structures of compounds **1**–**6**.

Upon a large scale fermentation of *S. albus* J1074/pCSG5033 (containing the intact *fls* gene cluster (Yang et al., 2015; Supplementary Figure 1) in a 40-L fermentator and the subsequent isolation with various chromatographic methods, FST C (**1**) was obtained as the major product, along with several new dimeric FSTs derived from non-enzymatic reactions (Huang et al., 2018). In addition, two new decaketide derivatives SEK43F (**2**) and fluoquinone (**3**), two known non-aketide-derived anthraquinones 2-acetylchrysophanol (**4**) (Abdelfattah, 2009) and 4-acetylchrysophanol (**5**) (Shaaban et al., 2007), and a previously synthesized compound 3,3′,4,4′,5,5′-hexamethyl-2,2′-dipyrrolylmethene (**6**) (Figure 1), were also isolated (Guseva et al., 2008; Lund and Thompson, 2014).

**Figure 2:**
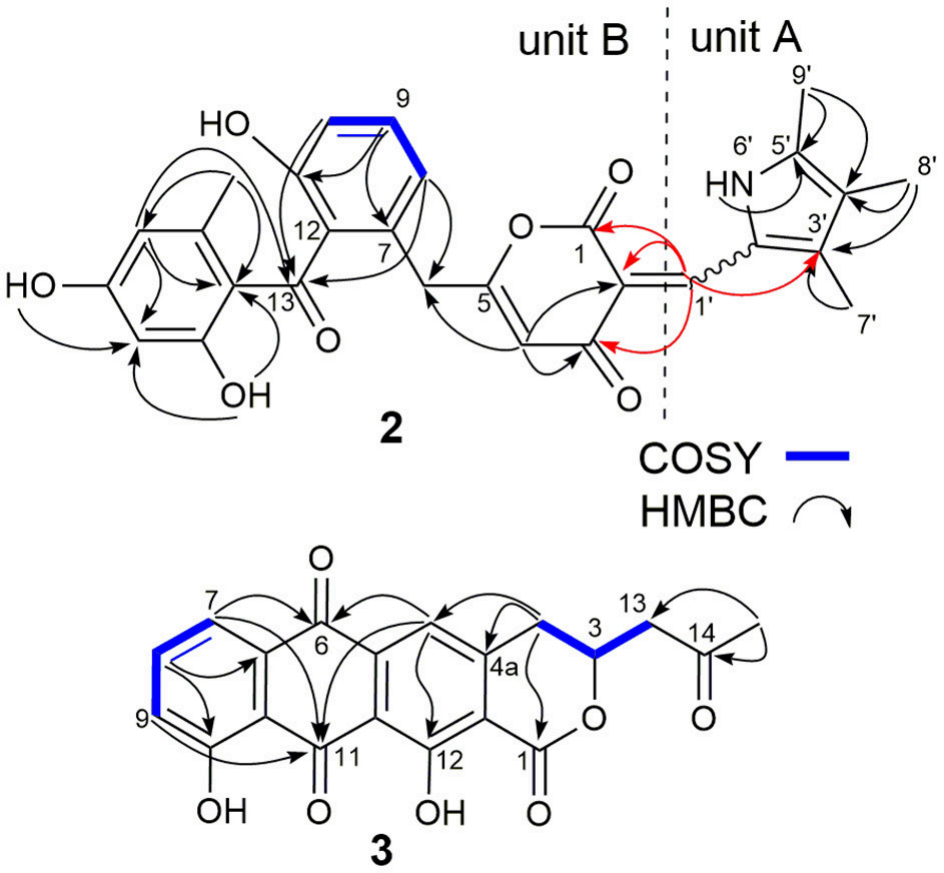
Selected key COSY and HMBC correlations of SEK43F (**2**) and fluoquinone (**3**). Key HMBC correlations connecting units A and B of **2** are in red.

SEK43F (**2**) was isolated as a yellow-green solid. The molecular formula of **2** was established to be C_28_H_25_NO_7_ (*m*/*z* 486.1557 [M - H]^−^, calcd for 486.1558) by high-resolution electrospray ionization mass spectroscopy (HRESIMS), suggesting 17 degrees of unsaturation. The NMR data of **2** (Table 1, Supplementary Figure 2) revealed the presence of four methyls, one methylene, seven sp^2^ methines, and 16 sp^2^ quaternary carbons. HMBC correlations from H_3_-7′ to C2′/C3′/C4′, H_3_-8′ to C3′/C4′/C5′, H_3_-9′ to C4′/C5′, and NH-6′ to C3′/C4′/C5′ revealed the presence of a 1,2,3,4-tetrasubstituted pyrrole moiety (unit A, Figure 2). The remaining NMR data of **2** (unit B, Figure 2) were highly similar to those for SEK43 (Meurer et al., 1997), including a characteristic 1,2,3-trisubstituted phenyl fragment (δ_H_ 6.82, 1H, d, *J* = 8.8 Hz; 7.24, 1H, dd, *J* = 8.2, 8.8 Hz; δ_H_ 6.83, 1H, d, *J* = 8.2 Hz; Table 1) and *meta* couplings spin system of H16 (δ_H_ 6.11, 1H, d, *J* = 2.3 Hz) and H18 (δ_H_ 6.07, 1H, d, *J* = 2.3 Hz) (Figure 2, Table 1). In addition, presence of the β-oxo-δ-lactone moiety was supported by HMBC correlations from H4 (δ_H_ 5.59) to C2/C3/C5, and from H1′ (δ_H_ 7.84) to C1/C2/C3 (Figure 2, Supplementary Figure 2). Finally, the two units A and B were linked by C2–C1′, which was supported by HMBC correlations (Figure 2, Supplementary Figure 2) from H1′ (δ_H_ 7.84) to C3′ and C1/C2/C3. However, the *Z*/*E* configuration of Δ^2,1′^ in **2** couldn't be determined solely by NMR data. Similar phenomena were observed for hybrubins (Zhao et al., 2016).

The molecular formula of fluoquinone (**3**) was determined to be C_20_H_14_O_7_ (*m*/*z* 365.0672 [M - H]^−^, calcd for 365.0667) by HRESIMS, requiring 14 degrees of unsaturation. The ^1^H, ^13^C, and HSQC NMR data for **3** displayed a singlet methyl, two sp^3^ methylenes, one sp^3^ methine, four sp^2^ methines, and 12 sp^2^ quaternary carbons (Table 1, Supplementary Figure 3). The ^1^H-^1^H COSY spectrum of **3** (Figure 2, Supplementary Figure 3, Table 1) showed a characteristic aromatic ABC spin system (δ_H_ 7.70/7.79/7.40) and a CH_2_-CH(O)-CH_2_ fragment (δ_H_ 3.31/4.12/5.02 and 3.09/3.13/5.02). HMBC correlations (Figure 2, Supplementary Figure 3) from H5/H7 to C6, from H8 to C10, together with four-bond HMBC correlations (Figure 2, Supplementary Figure 3) from H5/H7/H9 to C11, and from H5 to C12 supported the presence of a 1,8-dihydroxyanthraquinone moiety, highly similar to that of the fungal metabolite dermolactone (Gill and Gimenez, 1990; Cotterill et al., 1995). Comparison of NMR data revealed that **3** was different from dermolactone by the absence of a methoxy group at C8 (COSY correlations between H7/H8/H9 in **3**) and the presence of an additional acetyl group at C13 (HMBC correlations from H_3_-15 to C14/C13 in **3**). Thus, the planar structure of **3** was determined as shown in Figure 1. The optical rotation value of **3** ([α]25 D + 1.6, CHCl_3_; *c* 0.22) was quite different from that of the synthesized (*S*)-(+)-dermolactone ([α]22 D + 169.3, *c* 0.21) (Cotterill et al., 1995), but comparable to that of the naturally isolated dermolactone ([α]22 D + 22.0, CHCl_3_; *c* 0.07) (Gill and Gimenez, 1990). Natural dermolactone had been confirmed as a mixture of the (*S*)-(+)- and (*R*)-(-)-enantiomers (Cotterill et al., 1995). It was thus deduced that fluoquinone (**3**) should also contain a pair of racemic (*S*)-(+)- and (*R*)-(-)-enantiomers (almost in a ration of 1:1) due to its negligible optical rotation. Analysis of the ^1^H NMR and ^13^C NMR data of compounds **4**–**6** revealed that they were identical to 2-acetylchrysophanol (**4**) (Supplementary Figure 4, Supplementary Table 1) (Abdelfattah, 2009), 4-acetylchrysophanol (**5**) (Supplementary Figure 5, Supplementary Table 1) (Shaaban et al., 2007), and 3,3′,4,4′,5,5′-hexamethyl-2,2′-dipyrrolylmethene (**6**) (Supplementary Figure 6, Supplementary Table 1) (Guseva et al., 2008; Lund and Thompson, 2014).

### The biological evaluation

Compounds **1**–**6** exhibited negligible antibacterial activity against seven indicator strains: *S. aureus* ATCC 29213, *E. faecalis* ATCC 29212, *E. coli* ATCC 25922, *A. baumannii* ATCC 19606, *B. subtilis* SCSIO BS01, *Micrococcus Luteus* SCSIO ML01, and methicillin resistant *S. aureus* ATCC 43300. Compound **2** showed weak cytotoxic activities against four human cancer cell lines (SF-268, MCF-7, NCI-H460, and HepG-2) with IC_50_ values at the range of 36–57 μM (Table 2).

**Table 2:** Cytotoxic activities of SEK43F (**2**).

	**IC**_**50**_ **(**μ**M)**			

	**SF-268**	**MCF-7**	**NCI-H460**	**HepG-2**
**2**	56.46 ± 0.87	35.73 ± 1.45	44.62 ± 2.49	39.22 ± 3.00
**Cisplatin**	2.37 ± 0.17	2.94 ± 0.05	2.33 ± 0.17	1.39 ± 0.18

### Plausible biosynthetic pathway

**Figure 3:**
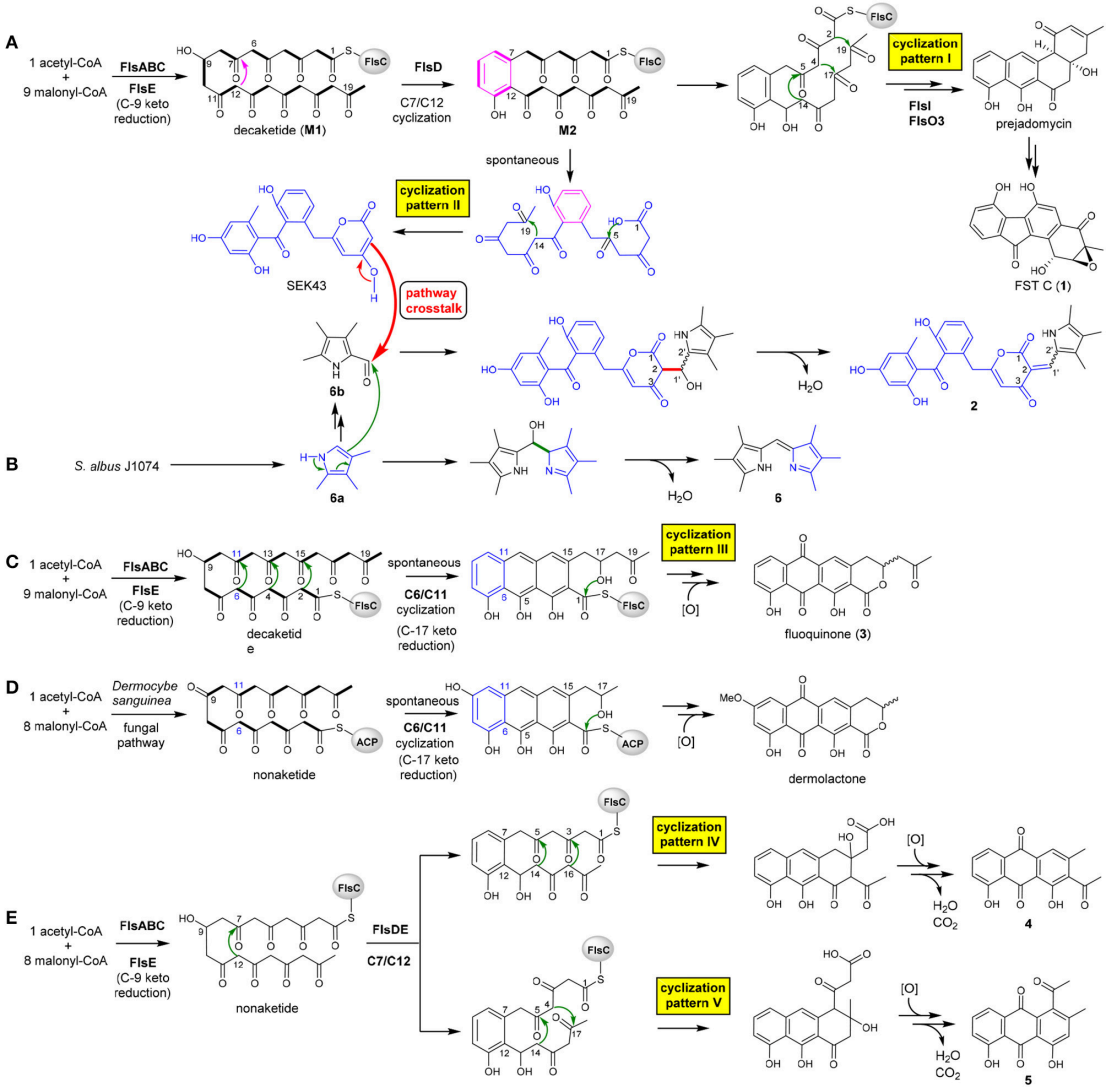
Proposed biosynthesis scheme leading to diversified APKs by taking 5 different cyclization strategies **(A–E)** and a pathway crosstalk between the host *S. albus* and the heterologous *fls* gene cluster **(A,B)**.

The heterologous expression of the intact *fls*-gene cluster from *M. rosaria* SCSIO N160 in *S. albus* J1074 afforded a set of APKs **1**–**5**. These aromatic compounds should be derived from the *fls* gene cluster-encoded enzymes because that the host *S. albus* J1074 is known for the absence of type II PKS gene clusters (Olano et al., 2014). Previously, a biosynthetic pathway of fluostatins has been proposed (Yang et al., 2015). Briefly, the type II PKS enzymes FlsA (the KS_α_ unit), FlsB (the KS_β_ unit) and FlsC (the dissociated ACP) assemble one unit of acetyl-CoA and 9 units of malonyl-CoA to generate a linear decaketide, which undergoes the FlsE-catalyzed C9 ketoreduction to give **M1** (Figure 3A). Subsequently, the aromatic cyclase FlsD catalyzes the “norm” C7/C12 first-ring cyclization to convert **M1** to **M2**. The downstream enzymes FlsI (a second cyclase analogous to JadI in jadomycin biosynthesis; Kulowski et al., 1999) and FlsO3 (oxygenases) converted **M2** to prejadomycin (**cyclization pattern I**: C7/C12, C5/C14, C4/C17, C2/C19), an established precursor to undergo oxidative modifications to produce diverse FST derivatives (e.g., **1**, Figure 3A) (Huang et al., 2018). Alternatively, **M2** could undergo spontaneous cyclization (**pattern II**: C7/C12, C14/C19, C1-OH/C5) to produce SEK43 (a shunt product often encountered in bacterial type II PKS pathways) (McDaniel et al., 1995; Hertweck et al., 2007), which is a key precursor leading to SEK43F (**2**). The tri-methylated pyrrole unit in **2**, which is also present in **6**, should be biosynthesized by the host strain, because that the production of **6** was also observed in *S. albus* J1074 harboring the void vector pSET152 (Supplementary Figure 1). We propose that **6** is produced by the coupling of two putative subunits **6a** and **6b** (Figure 3B), through a condensation reaction similar to RedH catalysis in the biosynthesis of undecylprodigiosin and hybrubins (Williamson et al., 2005; Zhao et al., 2016). The formation of **2** should be produced from a “pathway crosstalk” event via a condensation reaction by connecting C2 of SEK43 (generated by the heterologous *fls* type II PKS) and the aldehyde group of **6b** (produced by the host), either spontaneously or catalyzed by a host-derived RedH-like enzyme (Figures 3A,B) (Zhao et al., 2016).

It is intriguing to discover the anthraquinone derivative **3** in a bacterium. It was speculated that fundamentally different cyclization strategies were employed by bacterial and fungal type II PKSs to biosynthesize structurally similar APKs with fused-rings (Thomas, 2001, 2016). The first-ring cyclization modes were classified into F-mode for fungi to contain two intact acetate units, and S-mode for bacteria to contain three intact acetate units in the first cyclized phenol ring (Supplementary Figure 7) (Thomas, 2001, 2016; Zhou et al., 2010). This fundamental difference was reinforced by the discovery of three divergent first-ring cyclization strategies to converge in generating the same metabolite chrysophanol, namely F-mode (C6/C11) in fungi and plants (Bringmann et al., 2006), and S-mode (C5/C10) or S′-mode (C7/C12) in bacteria (Bringmann et al., 2006, 2009). Several bacterial fused-ring APKs were found as exceptions to be putatively derived from fungal F-mode cyclizations, such as TW93f and Tw93g (Shen et al., 1999), piloquinone (Polonsky and Lederer, 1963), murayaquinone (Gould et al., 1997), and haloquinone (Krone et al., 1981). However, it was recently discussed that evidence should be provided for these exceptions to either validate the structures (TW93f and Tw93g), or confirm the folding pattern by detailed labeling studies (Thomas, 2016). In contrast, the presence of the six-membered lactone ring in fluoquinone (**3**) strongly suggested that **3** should be derived from the first-ring cyclization via C6/C11 (**cyclization pattern III**: C6/C11, C4/C13, C2/C15, C17-OH/C1, Figure 3C). The fungal natural product dermolactone, highly similar to **3**, was confirmed to be derived from the F-mode of first ring cyclization (Figure 3D) (Gill and Gimenez, 1990). Thus, fluoquinone (**3**) represents an exception as a bacterial F-mode (fungal mode) to the F and S biosynthetic classifications of fused ring APKs (Thomas, 2001, 2016).

Compounds **4** and **5** were proposed to be derived from a common non-aketide, which was putatively assembled by FlsABC from one unit of acetyl-CoA and eight units of malonyl-CoA (Figures 3E,F). The discovery of **4** and **5** indicated that the CLF enzyme FlsB should exhibit a loose control in the chain length, allowing the formation of both decaketides (Figures 3A,C) and non-aketides (Figure 3E). The promiscuous chain length control has been reported for native type II PKS enzymes (McDaniel et al., 1993; Meurer et al., 1997; Shen et al., 1999), or can be altered by enzyme engineering (Burson and Khosla, 2000; Tang et al., 2003). Subsequently, the non-aketide was divergent in the third ring cyclization to provide two products **4** (**cyclization pattern IV**: C7/C12, C5/C14, C3/C16) and **5** (**cyclization pattern V**: C7/C12, C5/C14, C4/C17).

Although there are different cyclization patterns involved in the formation of **1**–**5**, the cyclization mechanisms are quite common and well-known in type II PKS biosynthesis: (i) enzyme-catalyzed or spontaneous cyclization via aldol condensation (Shen et al., 1999; Zhou et al., 2010), e.g., C7/C12, C6/C11, C5/C14, C4/C17, C4/C13, C3/C16, C2/C15, C2/C19, and C14/C19 (Figure 3); (ii) spontaneous cyclization via lactonization (Meurer et al., 1997), e.g., C1-OH/C5, C17-OH/C1 (Figure 3).

## Conclusion

This study expands our knowledge on the power of type II PKSs in diversifying APKs with the occurrence of five different cyclization patterns by a single set of type II PKSs. SEK43F (**2**) represents an unusual carbon skeleton resulting from a pathway crosstalk, in which a pyrrole-like moiety derived from the host *S. albus* J1074 is fused to an APK SEK43 generated from the heterologous *fls* type II PKSs. The occurrence of **3** highlights a bacterial APK that is exceptionally derived from a fungal F-mode first-ring cyclization.

## Author contributions

CZ and YZ designed the study protocol and directed the research. CH, WZ, and CheY performed compound isolation and structure determination. CH carried out the biological assays. ChuY conducted the *in vivo* genetic studies. CH, YZ, and CZ analyzed the data and wrote the manuscript.

### Conflict of interest statement

The authors declare that the research was conducted in the absence of any commercial or financial relationships that could be construed as a potential conflict of interest.
