# Supplementary material for: Marine Bacterial Aromatic Polyketides From Host-Dependent Heterologous Expression and Fungal Mode of Cyclization
Source: Front Chem. 2018 Oct 30;6:528. doi: 10.3389/fchem.2018.00528 (PMC6218434; doi:10.3389/fchem.2018.00528)
Supplement: Supplementary file 1 [file Table_1.DOCX]

Supplementary Material

Bacterial aromatic polyketides from host-dependent heterologous expression and fungal mode of cyclization

Chunshuai Huang^1,2^, Chunfang Yang^1^, Yiguang Zhu^1*^, Wenjun Zhang^1^, Chengshan Yuan^1^, and Changsheng Zhang^1,2*^

*** Correspondence:** Changsheng Zhang: [czhang2006@gmail.com](mailto:czhang2006@gmail.com); Yiguang Zhu: ygzhu@scsio.ac.cn

1. **Supplementary Figures and Tables**

## Supplementary Figures

**6** std.

**Supplementary Figure 1.** HPLC analysis of metabolite profile of expressing *fls*-gene cluster in *S. albus* J1074. (**A**) Genetic organization of the *fls*-gene cluster in *M. rosaria* SCSIO N160. (**B**) HPLC analysis of the fermentation extracts of heterologous expression of the *fls*-gene cluster in *S. albus* J1074 in the presence of 3% crude sea salts. (i) *S. albus* J1074/pCSG5033; (ii) *S. albus* J1074/pSET152; (iii) **6** standards. Structures for compounds **1**−**6** were shown in **Figure 1**. (**C** and **D**) Characterization of compound **6** produced by *S. albus* J1074 via UV comparison with the standard and LC-MS analysis.

(**i**). HRESIMS


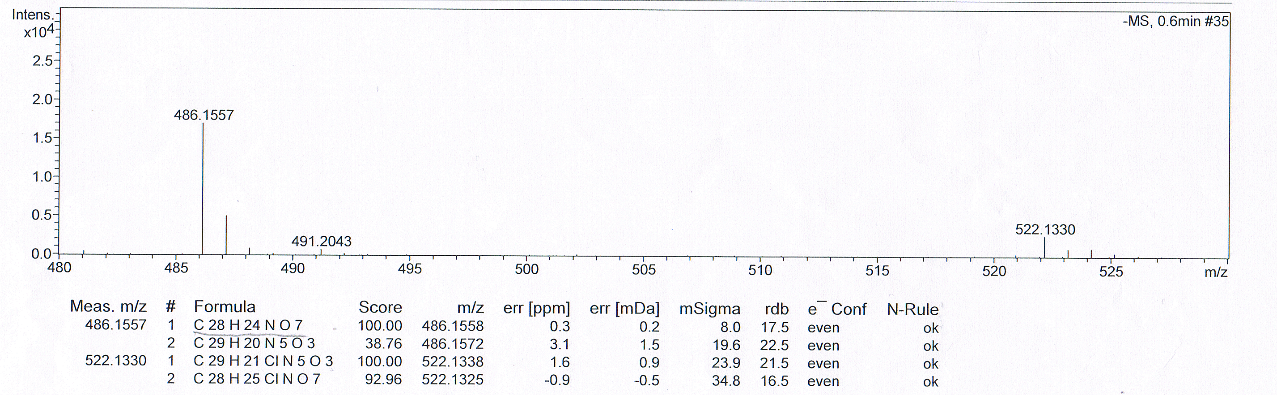


(**ii**). IR


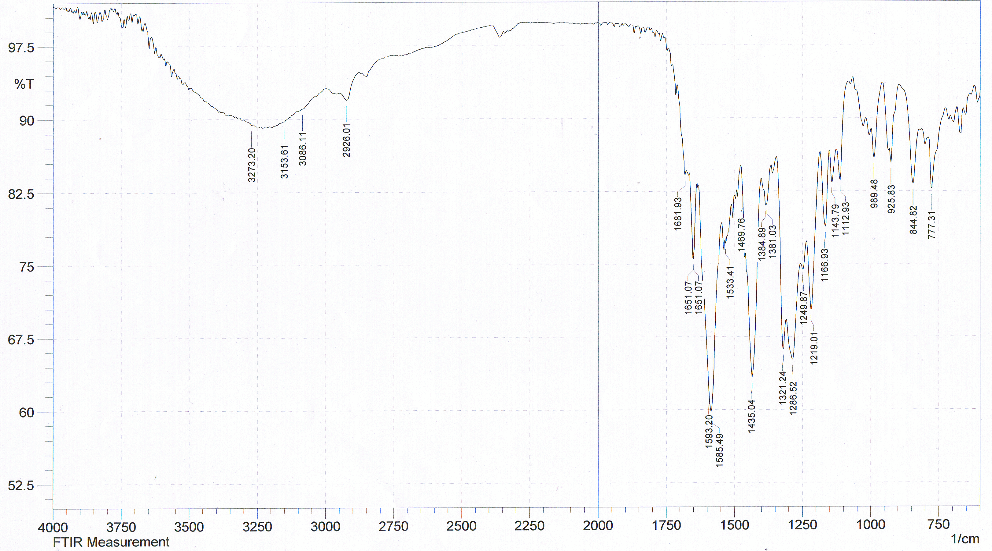


(**iii**). UV


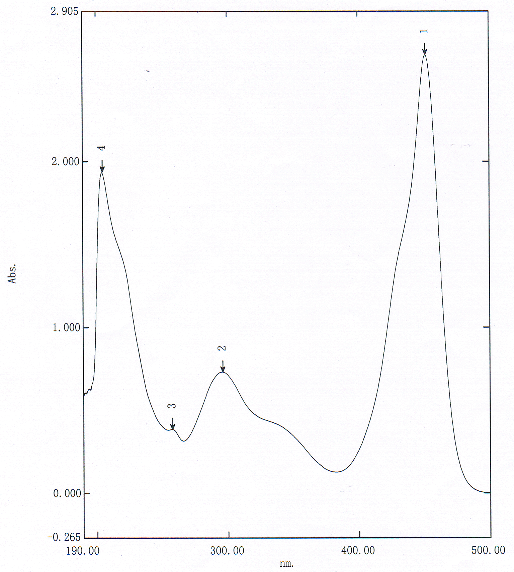


# Supplementary Figure 2. Spectroscopic data for SEK43F (2). (A): HRESIMS (i), IR (ii), and UV (iii) spectra of SEK43F (2).


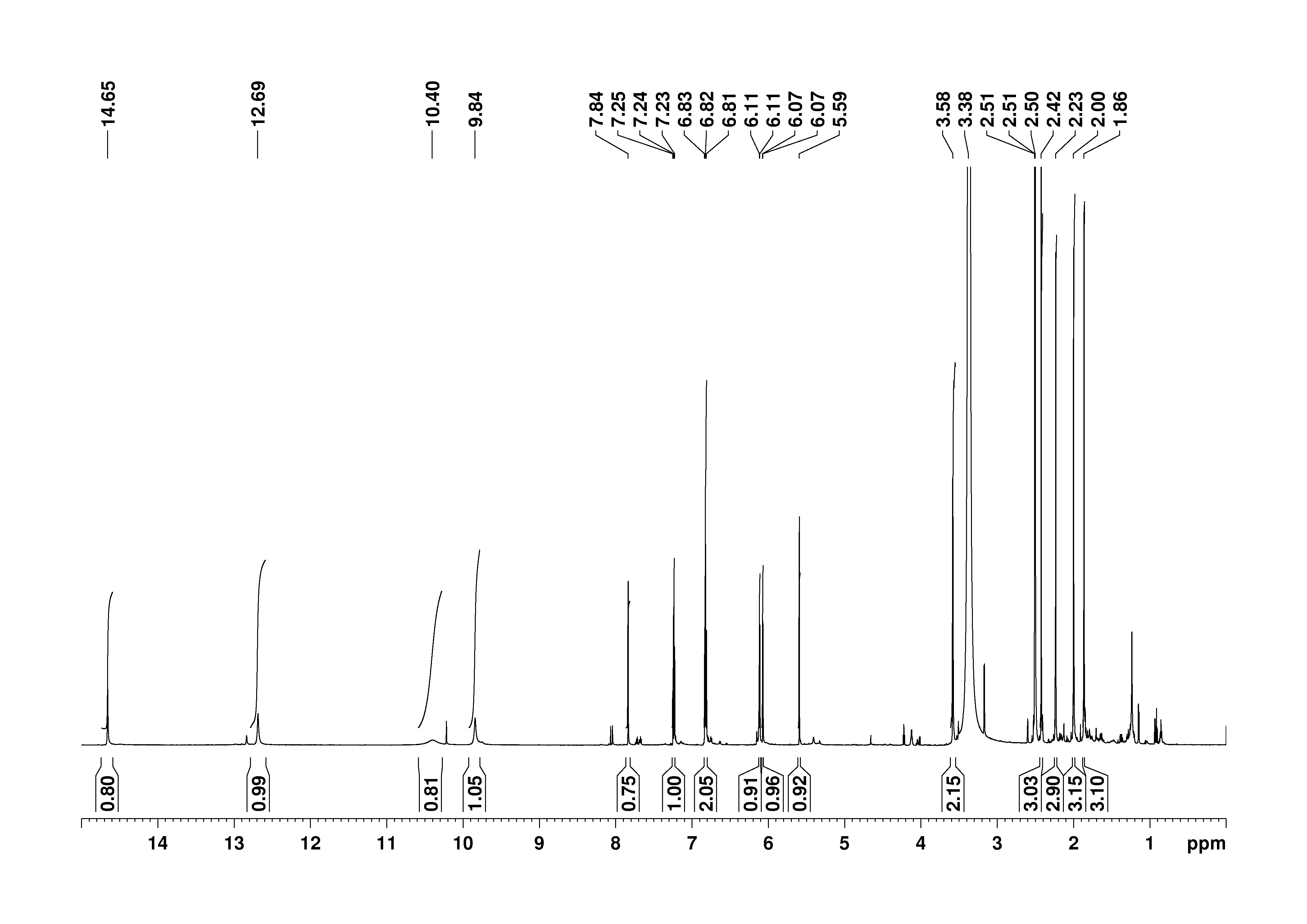


**Supplementary Figure 2.** Spectroscopic data for SEK43F (**2**). (continued) (**B**): The ^1^H NMR spectrum of SEK43F (**2**) in DMSO-*d*_6_.


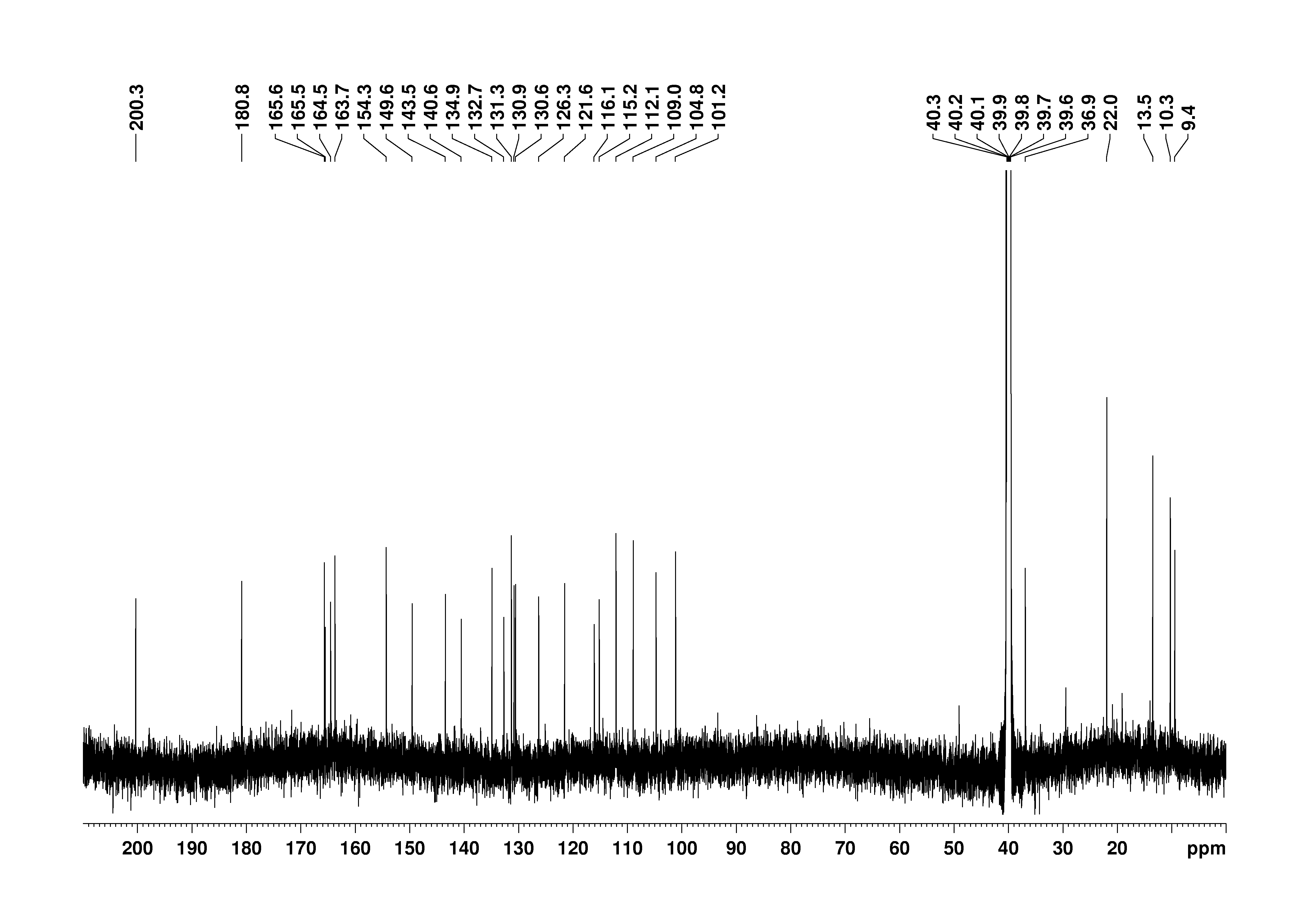


**Supplementary Figure 2.** Spectroscopic data for SEK43F (**2**). (continued) (**C**): The ^13^C NMR spectrum of SEK43F (**2**) in DMSO-*d*_6_.


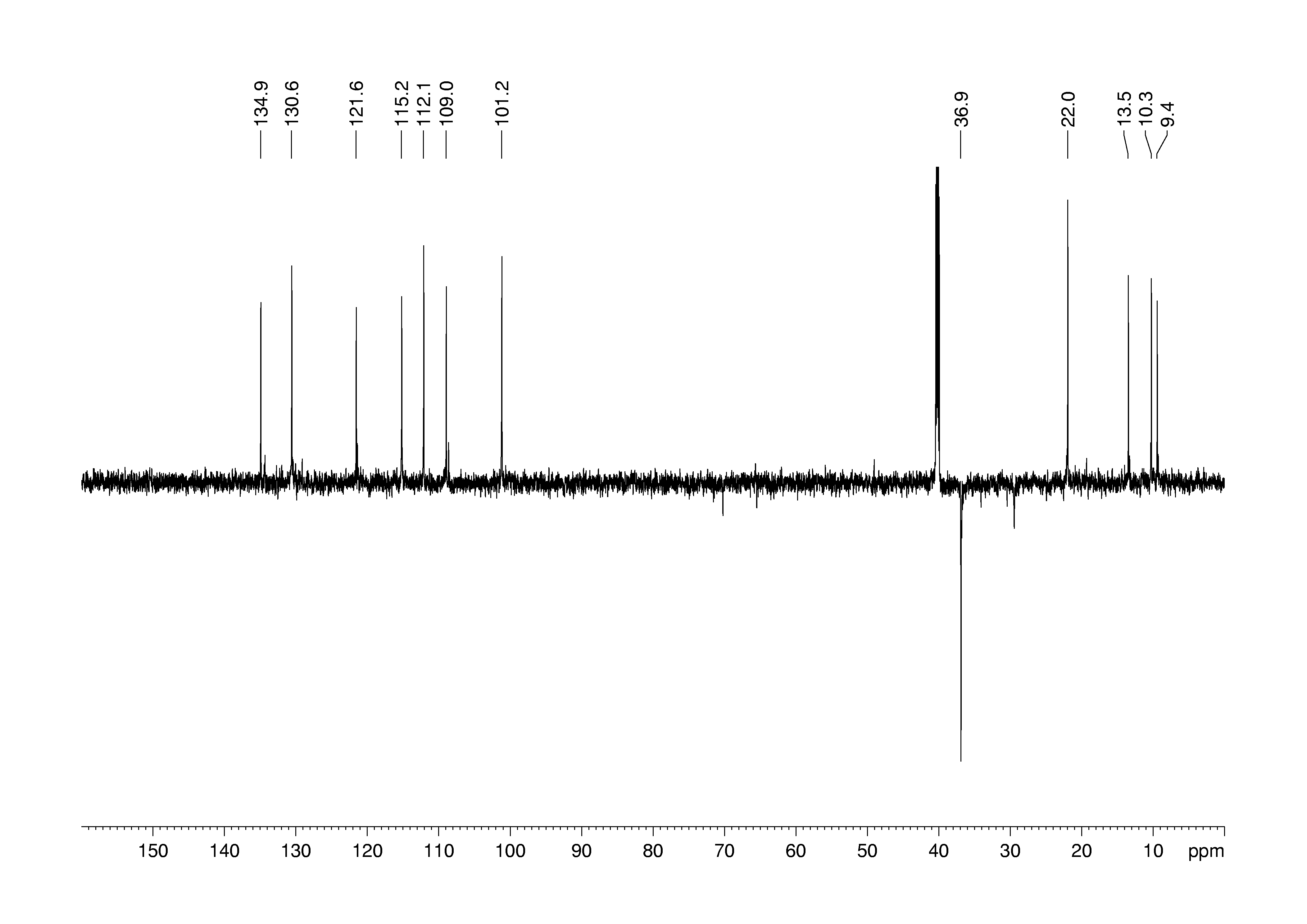


**Supplementary Figure 2.** Spectroscopic data for SEK43F (**2**). (continued) (**D**): The DEPT 135 NMR spectrum of SEK43F (**2**) in DMSO-*d*_6_.


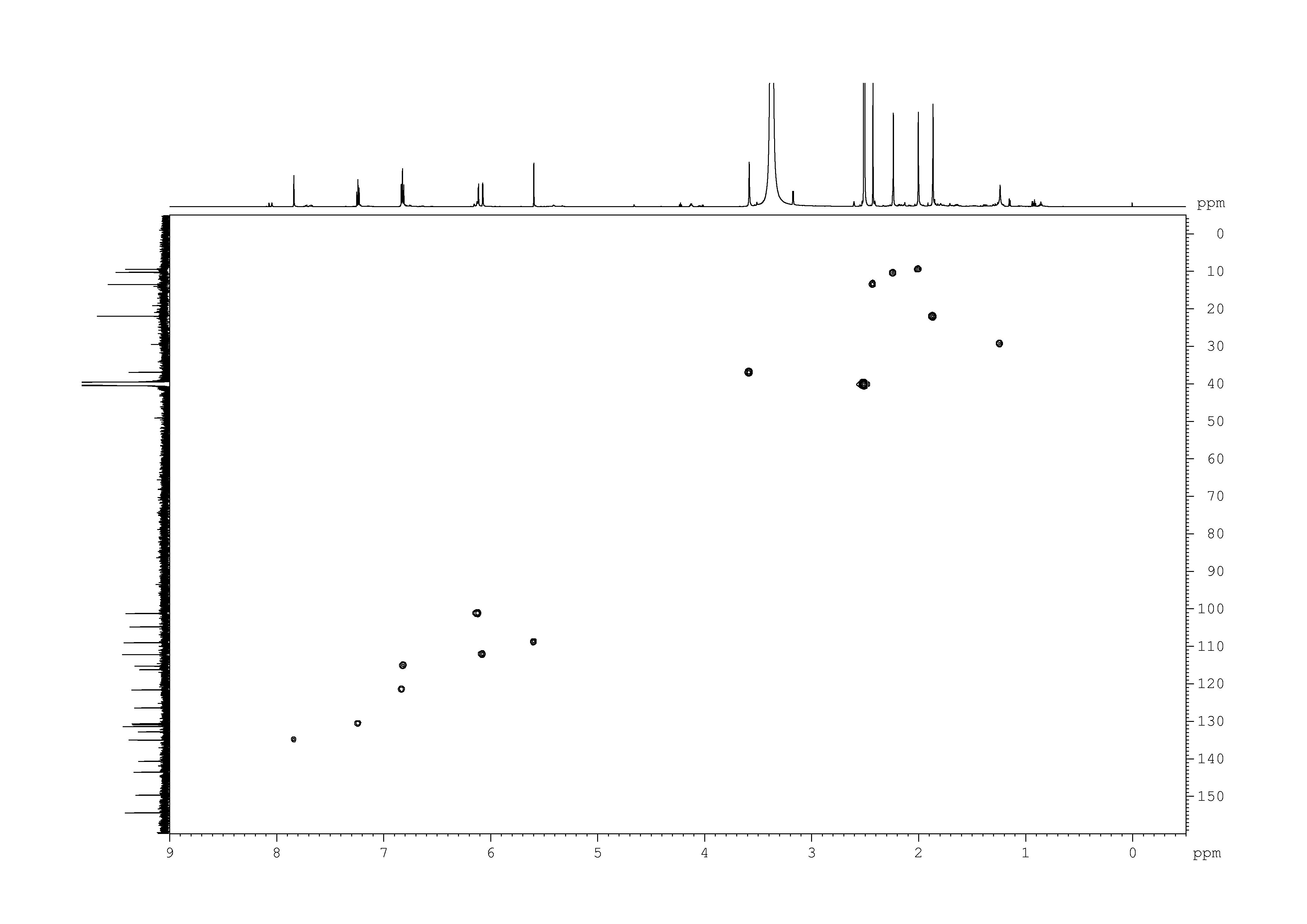


**Supplementary Figure 2.** Spectroscopic data for SEK43F (**2**). (continued) (**E**): The HSQC spectrum of SEK43F (**2**) in DMSO-*d*_6_.


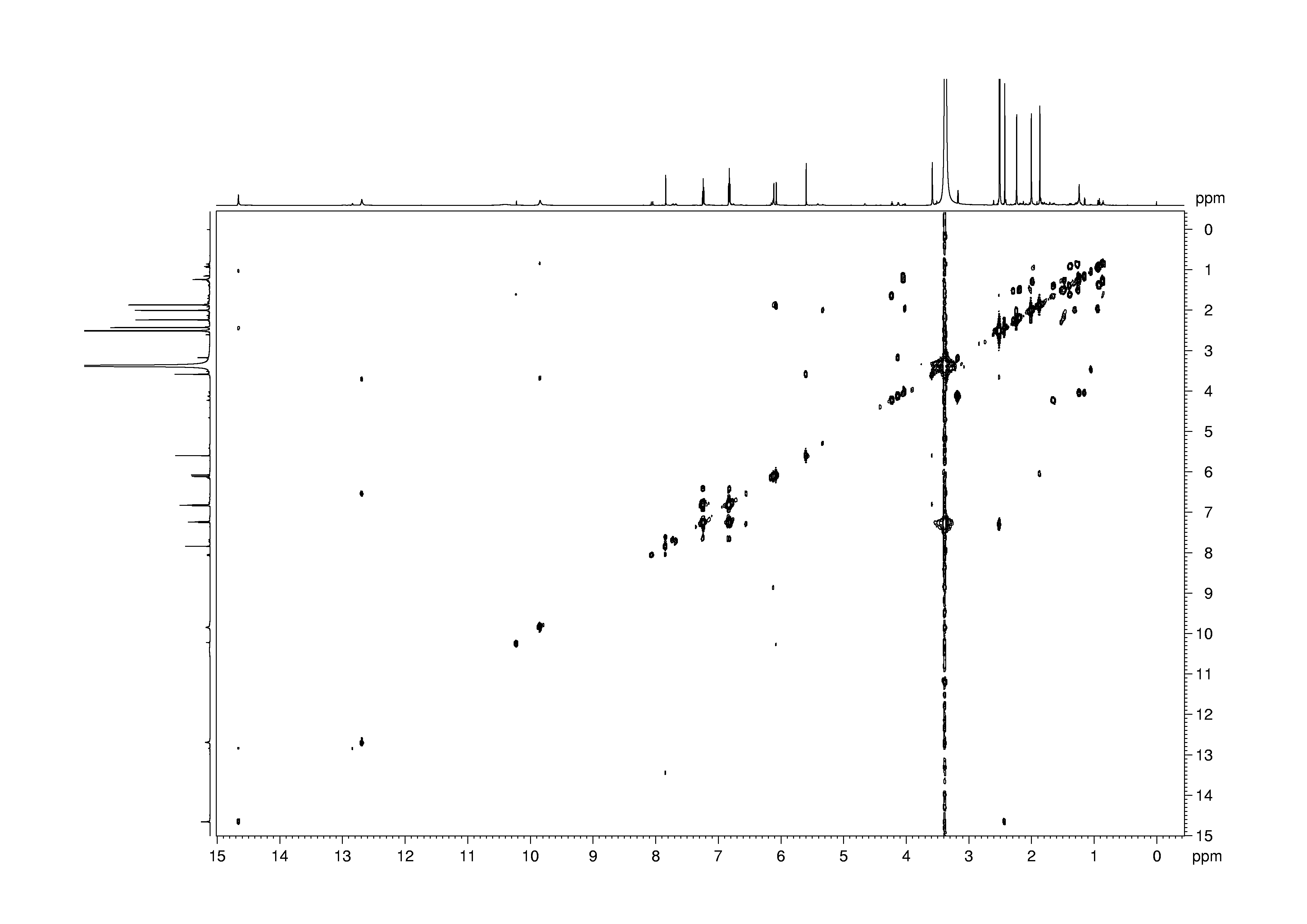


**Supplementary Figure 2.** Spectroscopic data for SEK43F (**2**). (continued) (**F**): The COSY spectrum of SEK43F (**2**) in DMSO-*d*_6_.


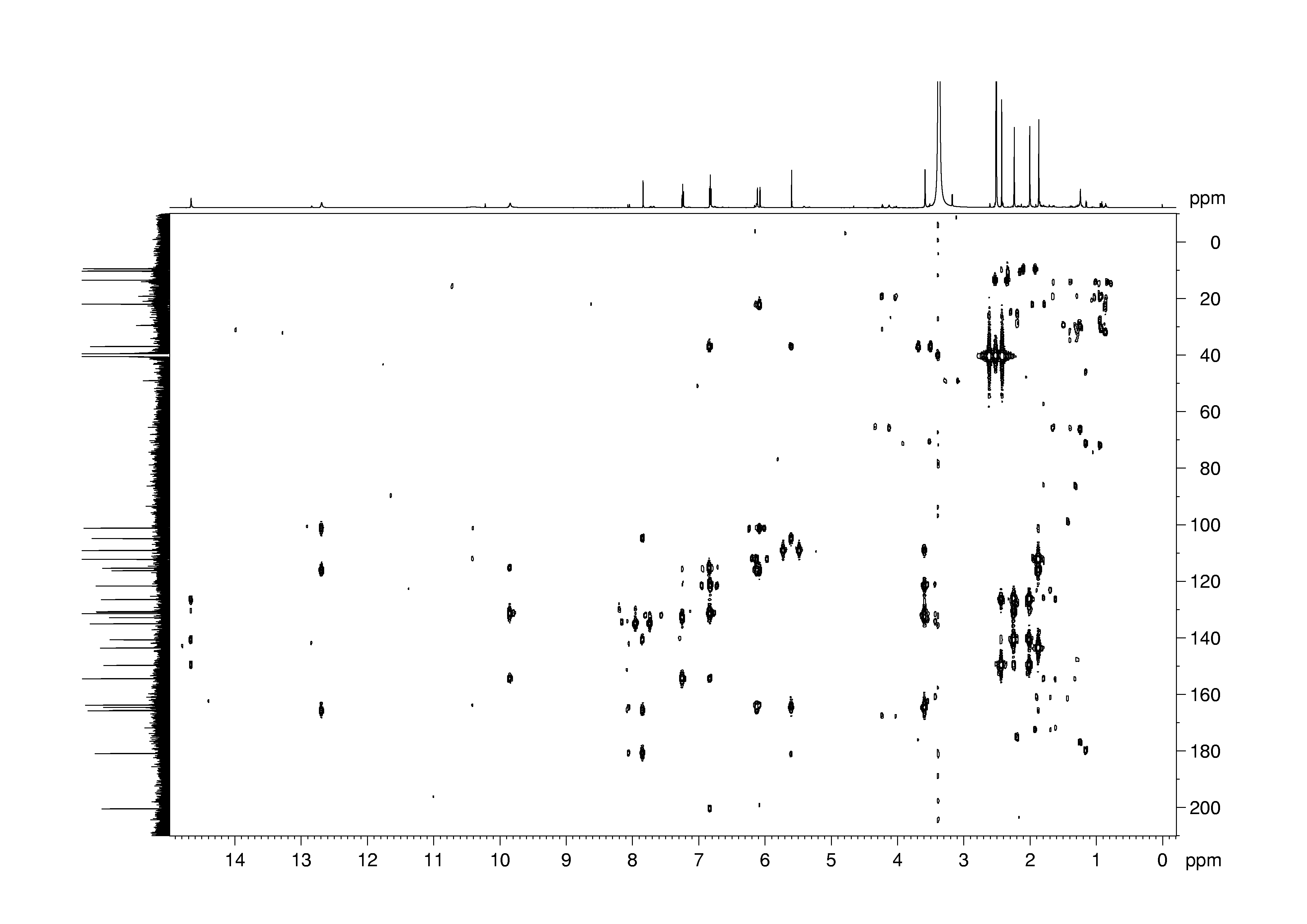


**Supplementary Figure 2.** Spectroscopic data for SEK43F (**2**). (continued) (**G**): The HMBC spectrum of SEK43F (**2**) in DMSO-*d*_6_.


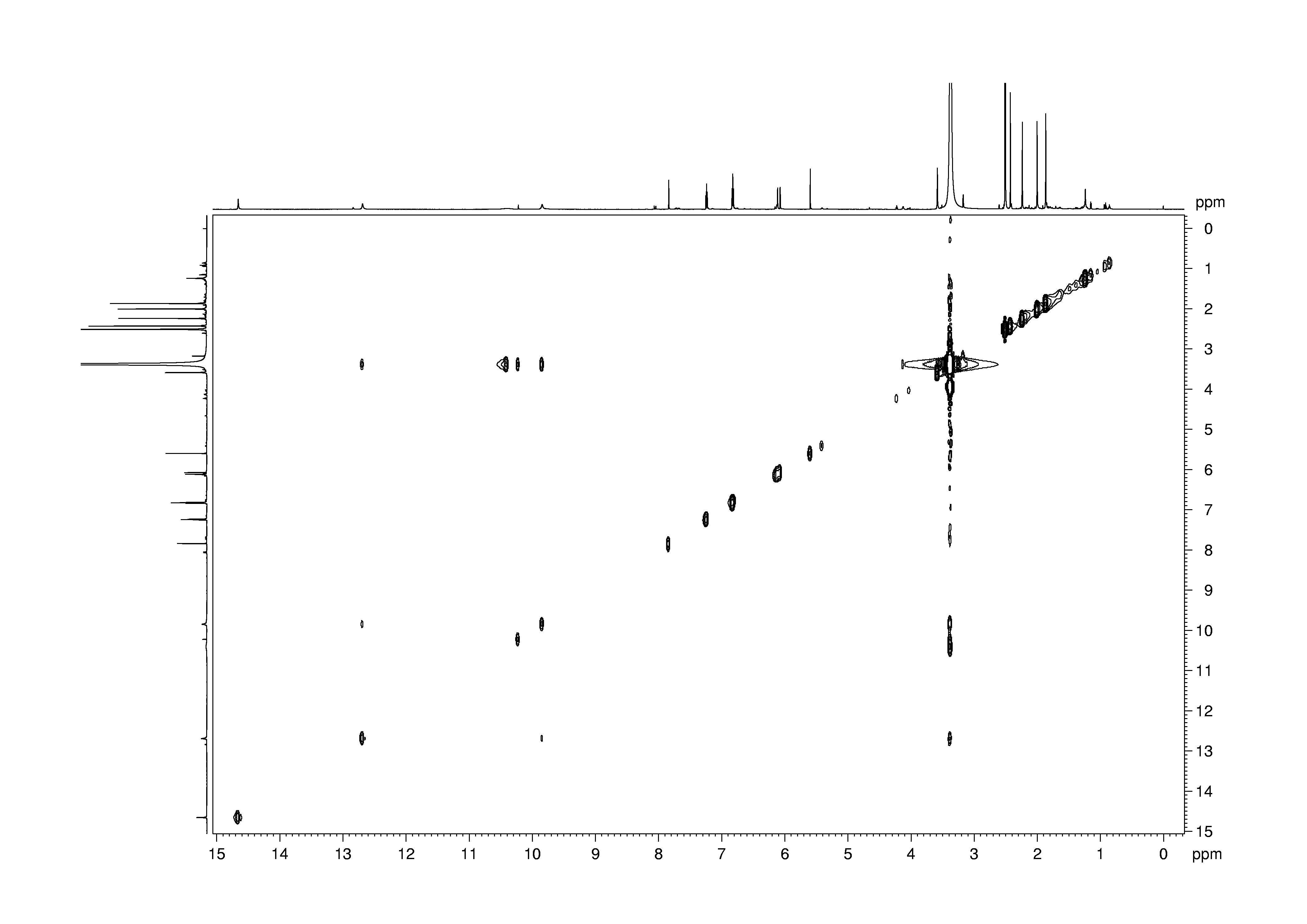


**Supplementary Figure 2.** Spectroscopic data for SEK43F (**2**). (continued) (**H**): The NOESY spectrum of SEK43F (**2**) in DMSO-*d*_6_.

(**i**). HRESIMS


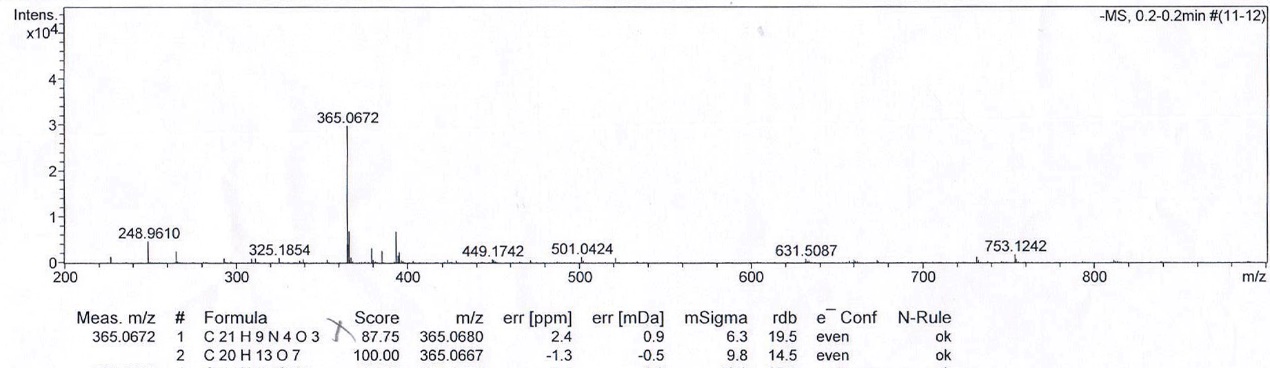


(**ii**). IR


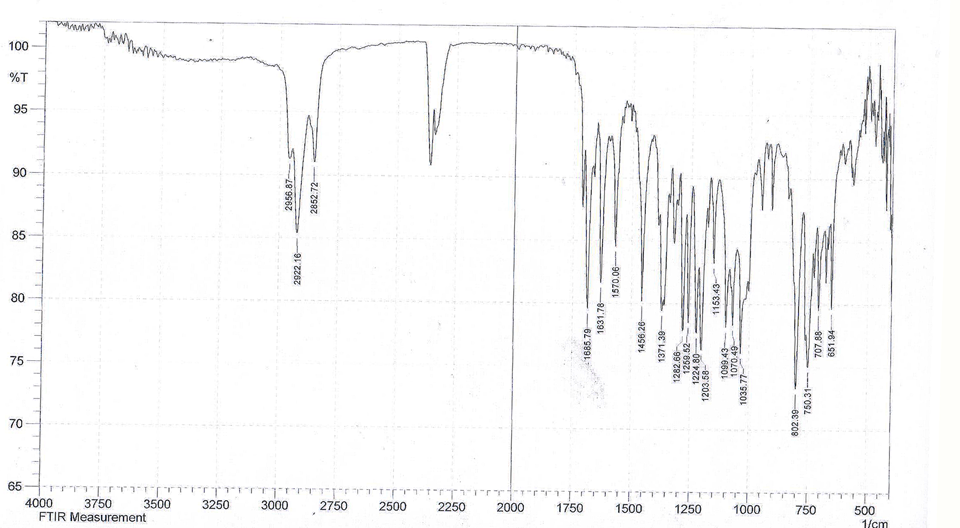


(**iii**). UV


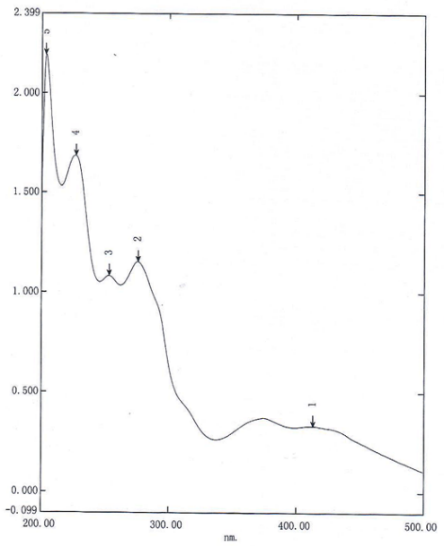


# Supplementary Figure 3. Spectroscopic data for fluoquinone (3). (A): HRESIMS (i), IR (ii), and UV (iii) spectra of fluoquinone (3).





**Supplementary Figure 3.** Spectroscopic data for fluoquinone (**3**). (continued) (**B**): The ^1^H NMR spectrum of fluoquinone (**3**) in DMSO-*d*_6_.





**Supplementary Figure 3.** Spectroscopic data for fluoquinone (**3**). (continued) (**C**): The ^13^C and DEPT 135 NMR spectra of fluoquinone (**3**) in DMSO-*d*_6_.





**Supplementary Figure 3.** Spectroscopic data for fluoquinone (**3**). (continued) (**D**): The HSQC spectrum of fluoquinone (**3**) in DMSO-*d*_6_.





**Supplementary Figure 3.** Spectroscopic data for fluoquinone (**3**). (continued) (**E**): The COSY spectrum of fluoquinone (**3**) in DMSO-*d*_6_.





**Supplementary Figure 3.** Spectroscopic data for fluoquinone (**3**). (continued) (**F**): The HMBC spectrum of fluoquinone (**3**) in DMSO-*d*_6_.

**Supplementary Figure 3.** Spectroscopic data for fluoquinone (**3**). (continued) (**G**): The enlarged HMBC spectrum of fluoquinone (**3**) in DMSO-*d*_6_.

**
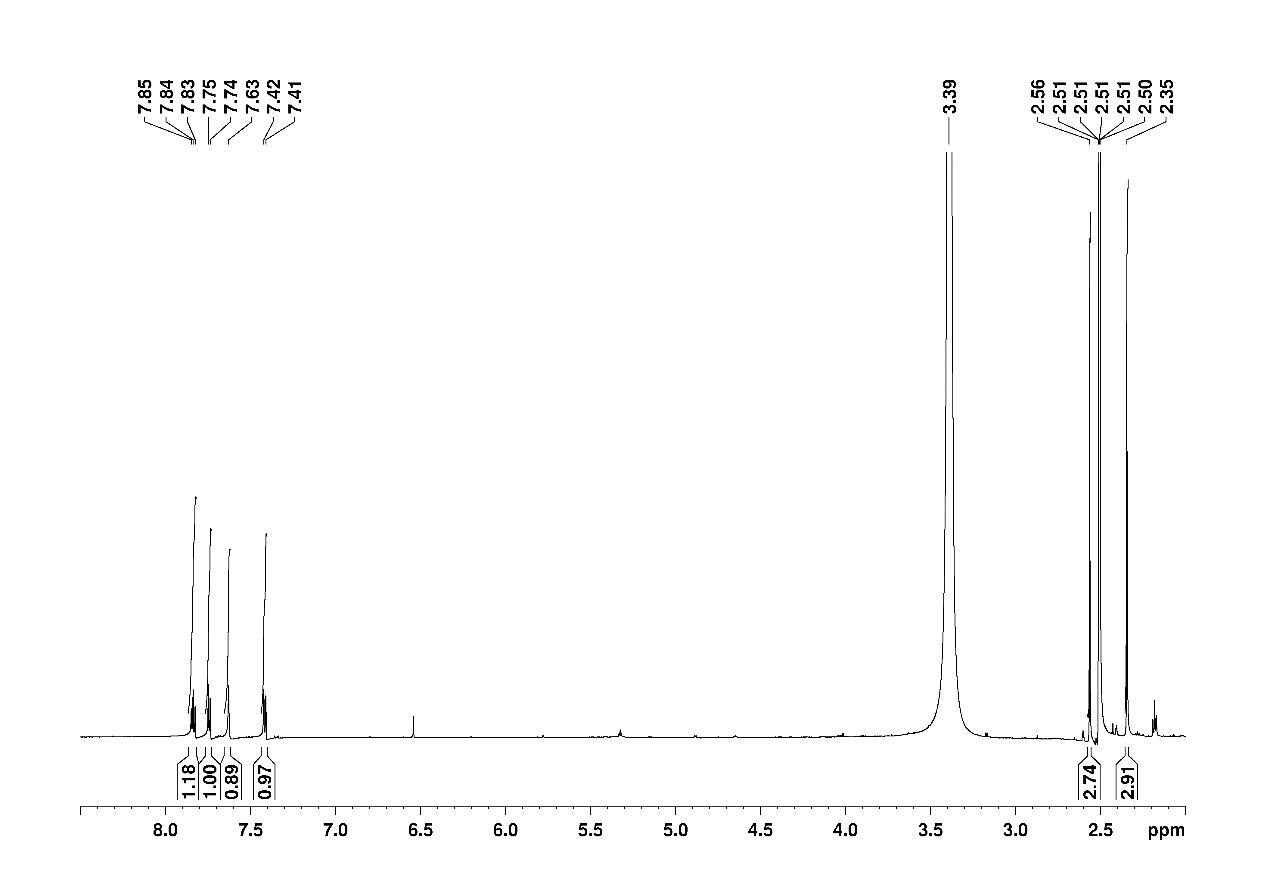
**

**A**


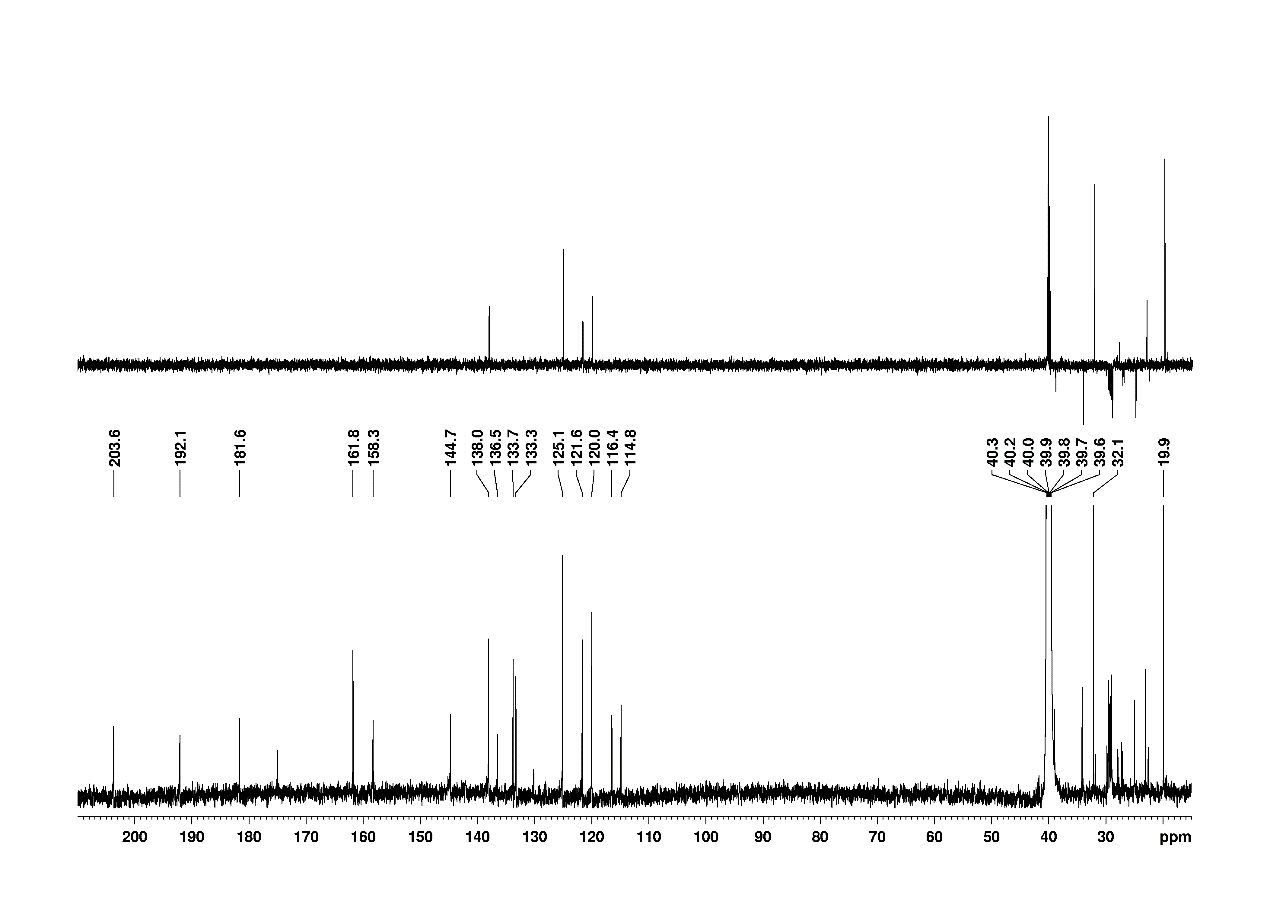


**B**

**Supplementary Figure 4.** Spectroscopic data for 2-acetylchrysophanol (**4**). (**A**): The ^1^H NMR spectrum of 2-acetylchrysophanol (**4**) in DMSO-*d*_6_. (**B**): The ^13^C and DEPT 135 NMR spectra of 2-acetylchrysophanol (**4**) in DMSO-*d*_6_.


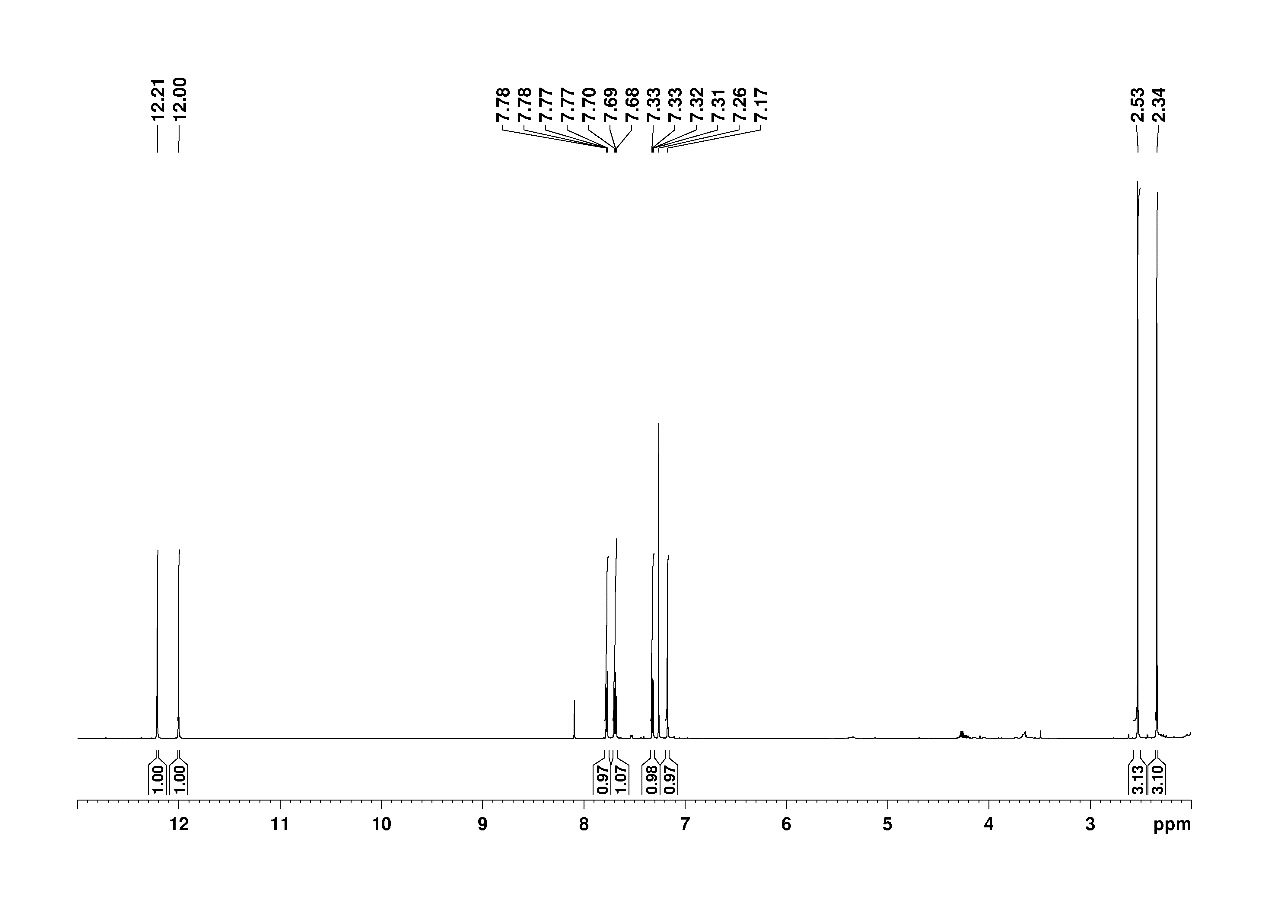


**A**


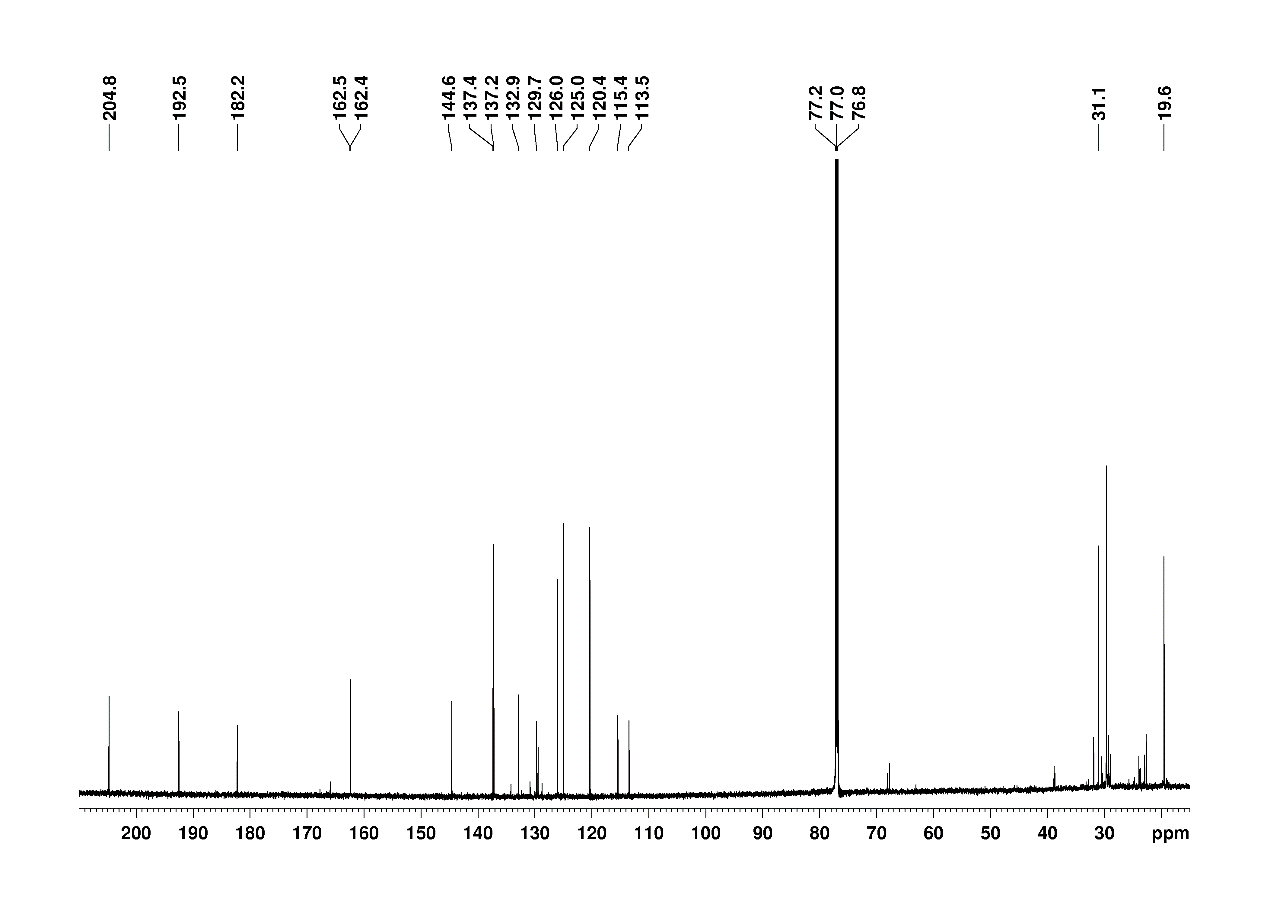


**B**

**Supplementary Figure 5.** Spectroscopic data for 4-acetylchrysophanol (**5**). (**A**): The ^1^H NMR spectrum of 4-acetylchrysophanol (**5**) in CDCl_3_. (**B**): The ^13^C NMR spectrum of 4-acetylchrysophanol (**5**) in CDCl_3_.

**
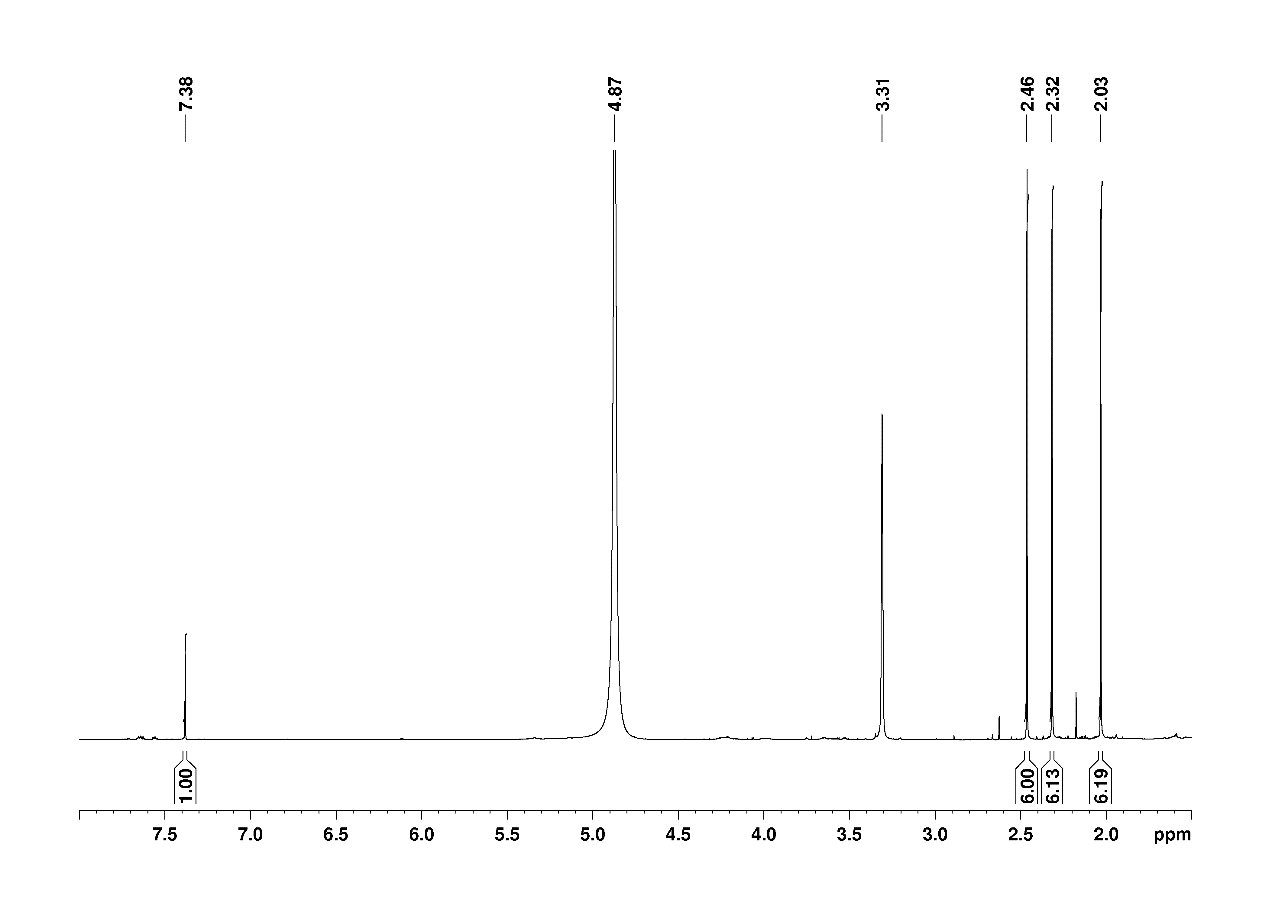
**

**A**


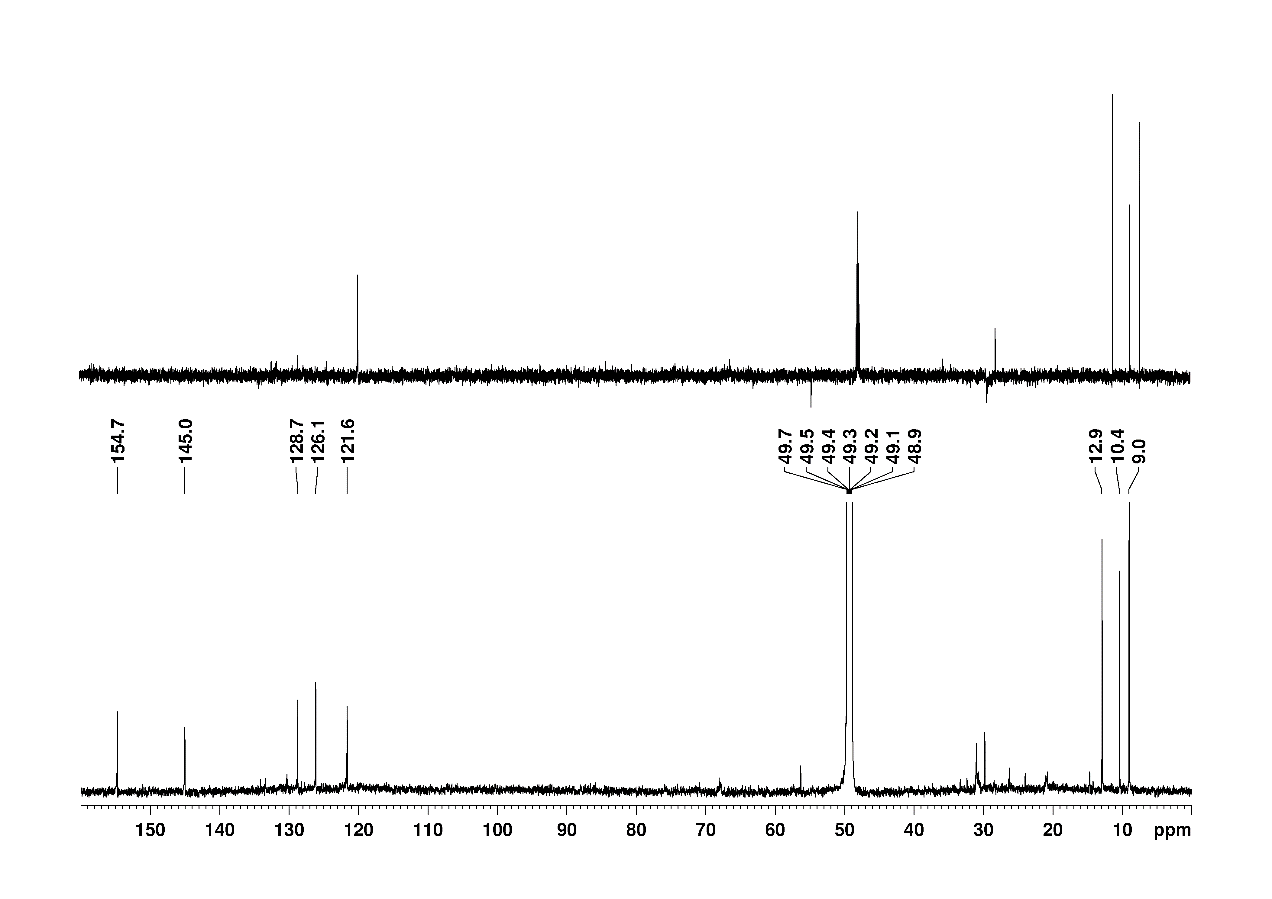


**B**

**Supplementary Figure 6.** Spectroscopic data for 3,3',4,4',5,5'-hexamethyl-2,2'-dipyrrolylmethene (**6**). (**A**): The ^1^H NMR spectrum of 3,3',4,4',5,5'-hexamethyl-2,2'-dipyrrolylmethene (**6**) in CD_3_OD. (**B**): The ^13^C and DEPT 135 NMR spectra of 3,3',4,4',5,5'-hexamethyl-2,2'-dipyrrolylmethene (**6**) in CD_3_OD.

# Supplementary Figure 7. The respective first-ring cyclization modes for fungi and bacteria aromatic polyketides. (A): Scheme for bacterial S-Mode of cyclization and fungal F-Mode of cyclization; (B): Examples for first-ring cyclization of F-mode (C6/C11) and S-mode (C7/C12).

## Supplementary Tables

**Supplementary Table 1.** ^1^H (700 MHz) and ^13^C (176 MHz) NMR data for compounds **4**−**6**.

| **4***^a^* | | |  | **5***^b^* | | |  | **6***^c^* | | |
| --- | --- | --- | --- | --- | --- | --- | --- | --- | --- | --- |
| Position | *δ*_C_*,* type | *δ*_H_*,* mult. (*J* in Hz) |  | Position | *δ*_C_*,* type | *δ*_C_*,* mult. (*J* in Hz) |  | Position | *δ*_C_*,* type | *δ*_H_*,* mult. (*J* in Hz) |
| 1 | 158.3*,* C |  |  | 1 | 162.5*,* C |  |  | 2 | 128.7*,* C |  |
| 2 | 136.5*,* C |  |  | 2 | 126.0*,* CH | 7.17*,* s |  | 3 | 145.0*,* C |  |
| 3 | 144.7*,* C |  |  | 3 | 144.6*,* C |  |  | 4 | 126.1*,* C |  |
| 3-CH_3_ | 19.9*,* CH_3_ | 2.35*,* s |  | 3-CH_3_ | 19.6*,* CH_3_ | 2.34*,* s |  | 5 | 154.7*,* C |  |
| 4 | 121.6*,* CH | 7.63*,* s |  | 4 | 137.2*,* C |  |  | 6 | 121.6*,* CH | 7.38*,* s |
| 4a | 133.3 C |  |  | 4a | 129.7*,* C |  |  | 7 | 10.4*,* CH_3_ | 2.32*,* s |
| 5 | 120.0*,* CH | 7.74*,* d (6.9) |  | 5 | 120.4*,* CH | 7.77*,* dd (1.0, 7.5) |  | 8 | 9.0*,* CH_3_ | 2.03*,* s |
| 6 | 138.0*,* CH | 7.84*,* dd (6.9, 8.1) |  | 6 | 137.4*,* CH | 7.69*,* dd (7.5, 8.4) |  | 9 | 12.9*,* CH_3_ | 2.46*,* s |
| 7 | 125.1*,* CH | 7.41*,* d (8.1) |  | 7 | 125.0*,* CH | 7.32*,* dd (1.0, 8.4) |  | 2′ | 128.7*,* C |  |
| 8 | 161.8*,* C |  |  | 8 | 162.4*,* C |  |  | 3′ | 145.0*,* C |  |
| 8a | 116.4*,* C |  |  | 8a | 115.4*,* C |  |  | 4′ | 126.1*,* C |  |
| 9 | 192.1*,* C |  |  | 9 | 192.5*,* C |  |  | 5′ | 154.7*,* C |  |
| 9a | 114.8*,* C |  |  | 9a | 113.5*,* C |  |  | 7′ | 10.4*,* CH_3_ | 2.32*,* s |
| 10 | 181.6*,* C |  |  | 10 | 182.2*,* C |  |  | 8′ | 9.0*,* CH_3_ | 2.03*,* s |
| 10a | 133.7*,* C |  |  | 10a | 132.9*,* C |  |  | 9′ | 12.9*,* CH_3_ | 2.46*,* s |
| 11 | 203.6*,* C |  |  | 11 | 204.8*,* C |  |  |  |  |  |
| 11-CH_3_ | 32.1*,* CH_3_ | 2.56*,* s |  | 11-CH_3_ | 31.1*,* CH_3_ | 2.52*,* s |  |  |  |  |
|  |  |  |  | 1-OH |  | 12.21*,* s |  |  |  |  |
|  |  |  |  | 8-OH |  | 12.00*,* s |  |  |  |  |

*^a^*Measured in DMSO-*d*_6_; *^b^*measured in CDCl_3_; *^c^*measured in CD_3_OD.
